# Functionalized boron nitride membranes with ultrafast solvent transport performance for molecular separation

**DOI:** 10.1038/s41467-018-04294-6

**Published:** 2018-05-15

**Authors:** Cheng Chen, Jiemin Wang, Dan Liu, Chen Yang, Yuchen Liu, Rodney S. Ruoff, Weiwei Lei

**Affiliations:** 1 Institute for Frontier Materials, Deakin University, Waurn Ponds, Victoria 3216 Australia; 2 Centre for Multidimensional Carbon Materials, Institute of Basic Science, and Department of Chemistry, Ulsan National Institute of Science and Technology, Ulsan 44919, Republic of Korea; 3 Department of Chemistry, Ulsan National Institute of Science and Technology (UNIST), Ulsan, 44919 Republic of Korea; 4 School of Materials Science and Engineering, Ulsan National Institute of Science and Technology (UNIST), Ulsan, 44919 Republic of Korea

## Abstract

Pressure-driven, superfast organic solvent filtration membranes have significant practical applications. An excellent filtration membrane should exhibit high selectivity and permeation in aqueous and organic solvents to meet increasing industrial demand. Here, we report an amino functionalized boron nitride (FBN) based filtration membrane with a nanochannel network for molecular separation and permeation. This membrane is highly stable in water and in several organic solvents and shows high transport performance for solvents depending on the membranes’ thickness. In addition, the FBN membrane is applicable for solute screening in water as well as in organic solvents. More importantly, the FBN membranes are very stable in acidic, alkaline and oxidative media for up to one month. The fast-flow rate and good separation performance of the FBN membranes can be attributed to their stable networks of nanochannels and thin laminar structure, which provide the membranes with beneficial properties for practical separation and purification processes.

## Abstract (web summary)

2D materials show promise for membrane filtration technologies, but their permeance to organic solvents is typically poor. Here, the authors prepare functionalized boron nitride membranes with high flux and high molecular separation performances in both aqueous solutions and organic solvents.

## Introduction

The high capital and operating costs of the separation, recovery, and disposal of organic solvents in chemical and pharmaceutical industries account for 40–70% of the total process cost due to reactions and high-value products in organic solvents at the end of the process^1,2^. In addition to conventional purification and separation processes, such as distillation, evaporation, adsorption, extraction, and chromatography, membrane processes have been steadily applied to industrial applications for 50 years to help meet economic, environmental and safety demands^3^. A new ideal membrane technology, the organic solvent nanofiltration (OSN) membrane, has been realized for more efficient solvent operations because of its excellent stability and high separation in organic solvents and good solvent permeance to improve the production process speed^1,3^. Meanwhile, several reports have highlighted that limited ultrahigh permeance membranes will not improve the separation process^4–8^. Therefore, membranes with a high solvent permeance and good separation performance have attracted interest from researchers for industrial applications.

Recently, two-dimensional (2D) nanomaterials (e.g., graphene^9,10^, exfoliated dichalcogenides^11,12^, layered metal oxides^13^, zeolites^14^, metal-organic framework (MOF) nanosheets^15^, and MXene^16^) have displayed promising performances in membrane science. Owing to their atomic thickness and controllable dimension units, 2D nanomaterials with unique nanopores and nanochannels possess extraordinary permeation properties^17,18^. Theoretically, the membranes of 2D nanomaterials function as a fence and allow some molecules to pass through smoothly while blocking others. Hence, the thickness and size of 2D nanomaterials play significant roles in the membrane permeation flux and selectivity. Based on the fabrication process, the membranes of 2D nanomaterials have two basic forms, nanosheets and laminar membranes^19^. By tailoring the in-plane and out-of-plane nanostructures, these membranes exhibit extraordinary performances for the separation of liquids^20^, gases^21^ and ions^22^. However, there are some issues impeding the use of 2D nanomaterials as further separating membranes. First, the permeance of reported membranes based on 2D nanomaterials for organic solvents is very poor, even for acetone, which has a low viscosity (0.32 centipoise, cP). Moreover, membranes based on 2D nanomaterials are commonly applied in limited solvents rather than organic and aqueous solvents, restricting their potential industrial applications. Graphene derivative membranes are reportedly unstable in water or under harsh chemical conditions and are not applicable as ideal separating membranes^23,24^. Finally, to improve the permeation flux and rejection selectivity, the thickness of some membranes has to be enhanced, increasing their cost and decreasing their effectiveness^11^. Therefore, to address these issues, a membrane based on hexagonal boron nitride (h-BN) could be an attractive and promising replacement for the current 2D nanomaterials because BN is chemically stable under harsh conditions, such as acidic and basic solutions^25,26^. However, until now, the use of h-BN membranes for organic solvent and water separation applications has not been reported because h-BN has a poor water dispersibility and cannot form membranes easily due to its hydrophobic property^27–31^. Hence, the fabrication of separation membranes based on h-BN still remains a challenge.

Herein, we developed ultrafast thin membranes based on 2D nanomaterials prepared using water-dispersible and few-layered h-BN flakes on commercially available microfiltration membranes. To create a water-dispersible h-BN solution, we functionalized the few-layered h-BN flakes (100–300 nm dimensions) with amino groups via a one-step exfoliation and functionalization process^32^. Typically, a FBN membrane with a thickness of 400 nm shows a pure solvent permeance as high as 5950 L m^−2^ h^−1^ bar^−1^ for acetone at room temperature. The membrane also shows a high water permeance performance of 2700 L m^−2^ h^−1^ bar^−1^ and a high durability in the long cycle test for up to 30 cycles by adjusting the thickness of the FBN membrane. This FBN-based membrane is also applicable to other organic solvents (e.g., methanol, acetone, and dimethylformamide). Furthermore, the prepared FBN membrane exhibits a high separation performance for dye molecules and a complete rejection of molecules larger than 5 nm in water. The membrane exhibits a high rejection rate for Congo red after soaking in strongly acidic (e.g., sulfuric acid), alkaline (e.g., potassium hydroxide), or oxidative (e.g., nitric acid) media for at least one month. More importantly, the FBN-based membranes also exhibit excellent separation performance in organic solvents, such as a >99% rejection rate for Au nanoparticles (5 nm) in ethanol (620 L m^−2^ h^−1^ bar^−1^) and a >99% rejection rate for Evans blue (1.2 nm × 3.1 nm) in methanol (560 L m^−2^ h^−1^ bar^−1^). This work demonstrates that the FBN membrane functions as a good candidate for high molecular separation with a high flux in both aqueous and organic solvents.

## Results

### Synthesis and characterization

**Fig. 1:**
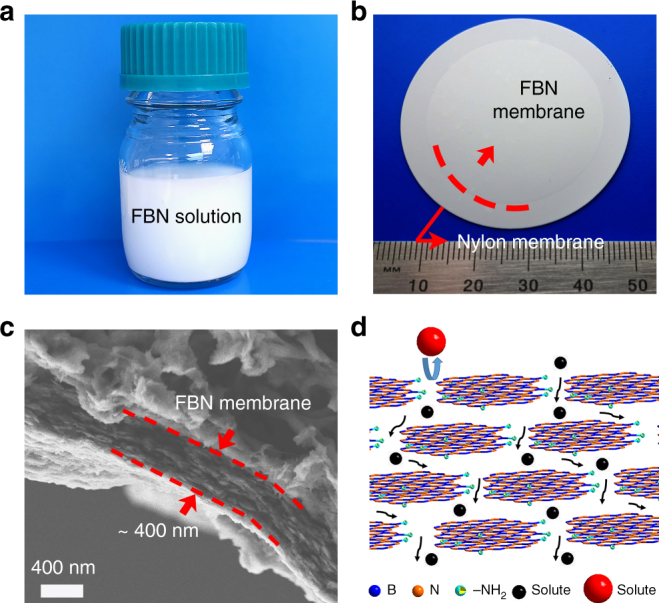
Preparation of the functionalized boron nitride membrane and solute separation mechanism. **a** A photograph of the prepared FBN dispersion in water. **b** A photograph of the prepared FBN on a commercial nylon membrane; the edge of the FBN membrane is shown with a red dotted line. **c** The cross-sectional SEM image of a FBN-0.4 membrane. **d** Illustration of the solute separation mechanism

The preparation of the FBN membranes and solute separation mechanism are shown in Fig. 1. The commercial h-BN was first exfoliated and functionalized by ball milling with urea, which was the functionalization agent^32^. Then, the FBN was dispersed in deionized (DI) water to form a white, stable aqueous dispersion (Fig. 1a). After dialysis cleaning, the as-prepared FBN dispersion was vacuum-filtered on a nylon membrane to obtain the FBN membrane (Fig. 1b). Interestingly, the thickness of the FBN membranes can be facilely adjusted by filtering different amounts of the FBN dispersion (Fig. 1c and Supplementary Fig. 1). We denote the membranes as FBN-*X*, where *X* is the thickness of the FBN membrane depending on the scanning electron microscopy (SEM) results (*X* = 0.4, 0.7, 1, 1.5, 2, and 8 μm). The choice of a nylon membrane as a supporting membrane is due to its stable structure in both acidic and alkaline conditions (Supplementary Fig. 2 and 3)^33–35^. Meanwhile, the nylon membrane possesses a large pore size of 0.2 μm, which will not influence the permeance of the FBN membranes. As observed by SEM (Fig. 1c), the thickness of the FBN-0.4 membrane is nearly 400 nm. In addition, the SEM image highlights the layer-by-layer-laminated structure in the cross section of the membrane. Notably, the laminated structure produces channels for small molecule shuttling^16^. The potential solute separation mechanism shows that small molecules can pass through the nanochannels of the FBN membrane, but the larger molecules are blocked (Fig. 1d).

**Fig. 2:**
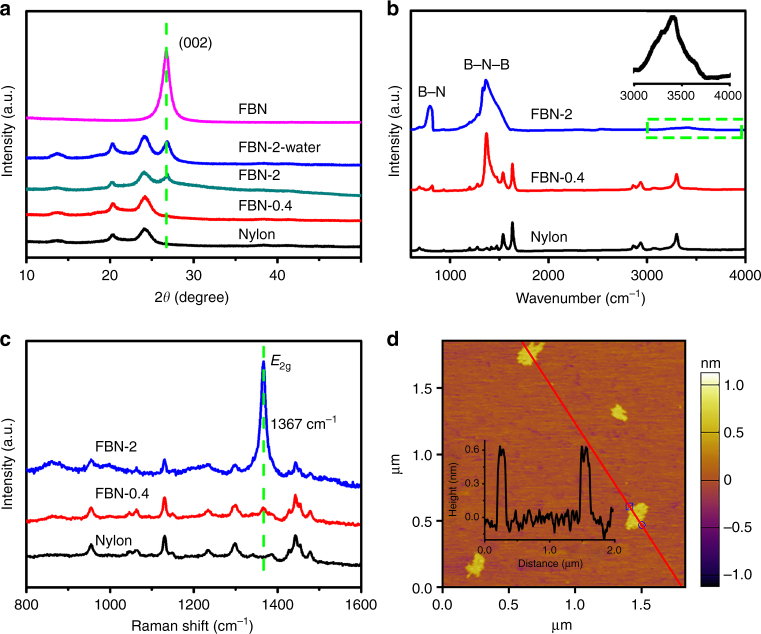
The structure of the functionalized boron nitride membranes. **a** XRD patterns of the FBN membrane on nylon. **b** Raman spectra of the FBN membrane on nylon. **c** FTIR-ATR images of the FBN membrane on nylon. **d** AFM of the FBN flakes, and the inset shows the corresponding height profile

The basic structural characterizations of the FBN membranes were performed using X-ray diffraction (XRD), Raman spectroscopy, attenuated total reflectance-Fourier transform infrared absorbance (FTIR-ATR) spectroscopy, and atom force microscopy (AFM). The characteristic FBN peaks linearly increase with the loading thickness. Figure 2a shows the XRD patterns of the prepared FBN membranes with different thickness. Compared with the pure nylon membrane, a characteristic diffraction peak can be observed at 26.8° as the FBN thickness increases, and the peak arises from the (002) plane of the FBN (Fig. 2a and Supplementary Fig. 4). The same diffraction peak position (002) of FBN samples indicates that the interlayer spacing of the FBN remains unchanged. Therefore, the molecular sieving occurs between the intra-layer spacing rather than the interlayer spacing^26^. The FTIR-ATR spectra show the functional groups of the prepared FBN membrane. The FTIR-ATR spectra (Fig. 2b and Supplementary Fig. 5) exhibit a strong absorption at 1380 cm^−1^ and a vibration peak centered at approximately 780 cm^−1^ due to the intrinsic BN functional groups, out-of-plane B–N–B bending vibration and in-plane B–N stretching vibration, respectively^36,37^. Furthermore, a broad peak exists from 3000–3600 cm^−1^ in the inset due to the N–H– (approximately 3250 cm^−1^) and O–H– (approximately 3410 cm^−1^) stretching vibrations from the fabricating process^38,39^. The appearance of the N–H stretching vibrations indicates successful functionalization of amino group. The amino group allows the FBN flakes to be stable at high concentrations in aqueous media^32^. The amino group can also be analyzed via an X-ray photoelectron spectroscopy (XPS) experiment. As shown in Supplementary Fig. 6a, the main peak at 190.4 eV is ascribed to the B–N bonds in FBN, and a shoulder peak at 191.6 eV is ascribed to the B–O bonds. The B–O bonds may come from the pristine BN, the fabrication process, or air moisture^38^. In Supplementary Fig. 6b, the main peak at 398.1 eV in the N 1 s spectrum corresponds to the N–B bonds of BN, while the satellite peak at 399.1 eV corresponds to the N–H bonds^40^. This confirms the amino group on the FBN. The Raman spectra (Fig. 2c and Supplementary Fig. 7) further demonstrate that the FBN membranes exhibit an intrinsic *E*_2g_ vibration peak at 1367 cm^−1^^41,42^, which gradually increases as the thickness of FBN increases. Therefore, all the XRD, FTIR-ATR, and XPS results confirmed that the FBN was functionalized with amino groups.

As shown in Fig. 2d, the obtained AFM pattern reveals that the FBN lamina exhibits a relatively uniform lateral size of approximately 100–300 nm. After the lateral membrane is assembled, the stacked FBN flakes can generate random nanochannels because of the inevitable slits along the edges of the sheets. According to recent literature^20,43,44^, three parameters affect the structure of nanochannels, namely, the height of the nanochannels, the lateral size of the individual flakes, and the gap distance between the ends of the flakes. The morphology of the FBN (Fig. 2d) confirms that the small FBN flakes are important in the fabrication of high-performance membranes and create more transport paths and channels for solvents. Furthermore, the SEM image of the FBN is shown in Supplementary Fig. 8, and the image shows the lateral dimensions range from ~100 nm to ~300 nm^45^. Most FBN flakes contain 2–4 layered BN flakes with a size area of 0.01–0.06 μm^2^. Thus, the small lateral FBN lamina creates more nanochannels for molecules to pass through.

### Membranes for solvent permeation

**Fig. 3:**
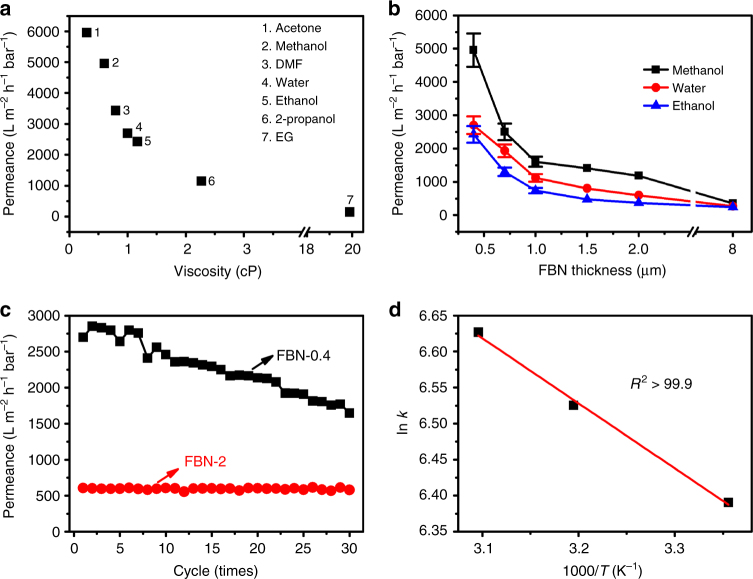
Pure solvent permeance of the functionalized boron nitride membranes. **a** The plot of the solvent filtration ability versus the viscosity of different solvents (EG is ethylene glycol) through FBN-0.4, and **b** the effect of the membrane thickness on the filtration. **c** The water permeation performance of the FBN-0.4 and FBN-2 membranes with 30 periodic operations of water filtration, and **d** the Arrhenius plot of the water permeation rate (ln *k*) versus inverse temperature (1000/T) for FBN-2

The permeance of various pure solvents through the prepared FBN membranes was investigated by passing 40 mL of the solvents through the membranes at room temperature under a pressure of 1 bar. Water and methanol were selected to test the leakage of FBN in the solution (Supplementary Fig. 9). Operators should be very careful when using the thin FBN membranes in experiments. As displayed in Fig. 3a and Supplementary Fig. 10, as the viscosity decreases, the FBN membrane exhibits an increasing permeance ability. Surprisingly, acetone passed through the FBN-0.4 membrane (≈400 nm in thickness) with an unprecedented value of 5950 L m^−2^ h^−1^ bar^−1^. Owing to the low viscosity of the solvents and the small sizes of the FBN flakes, the FBN membrane can be applied as the highly active membrane. However, for 2-propanol, an organic molecular similar in size to acetone, the permeance is 1140 L m^−2^ h^−1^ bar^−1^ due to its much higher viscosity (2.37 cP). This indicates that the solvent’s viscosity, molecular size, and membrane microstructure all contribute to the permeance performance. Figure 3b further depicts how the thickness of the membrane affects the solvent flux performance. Logically, a thicker membrane has a longer route that solvents must pass through. Interestingly, even though the thickness of the FBN-2 membrane is nearly 2000 nm, the membrane still has a high pure solvent permeance (1660 L m^−2^ h^−1^ bar^−1^) for acetone (Supplementary Fig. 10). Importantly, the FBN-2 membrane shows very stable physical and chemical property after filtration (Supplementary Fig. 11).

In addition, Fig. 3c plots the rate versus the number of operation cycles in water. The thinner membrane is more easily affected by water passing through, while the thicker membrane shows more stability. The FBN-2 membrane is quite stable (620 L m^−2^ h^−1^ bar^−1^) in water, even after 30 periodic operations of filtration. To further investigate the water transportation process, the molecular permeation rate was tested at 298 K, 313 K and 323 K, separately. The Arrhenius activation energy for water filtration through the prepared membrane was calculated by fitting the permeation rate (Fig. 3d)^46^. After fitting the speed (the linear fitting coefficient is 0.999) versus the operating temperature curve, the *E*_a_ value is 7.41 kJ/mol, indicating that the FBN membrane has a higher water flux ability compared to that of the graphene oxide membrane (*E*_a_ = 11.12 kJ/mol)^47^.

### Membranes for molecular separation

**Fig. 4:**
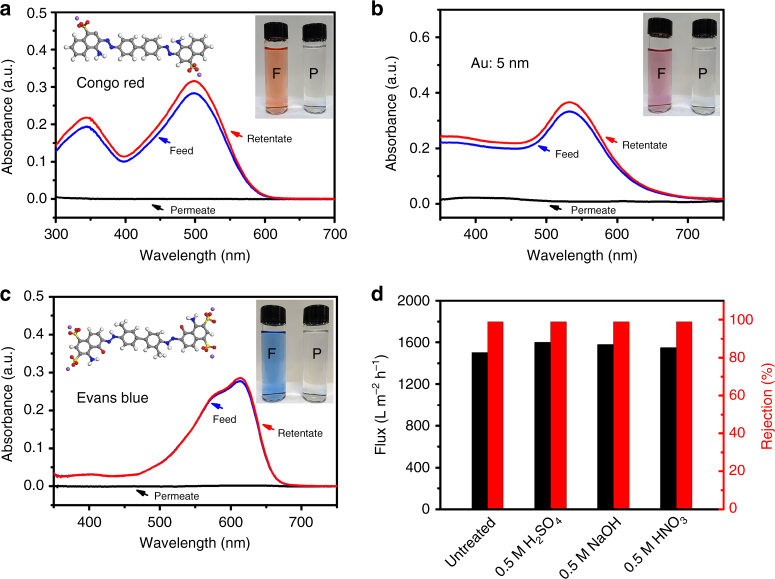
Molecular separation through the functionalized boron nitride membranes. **a** UV-Vis absorption spectra of an aqueous solution of Congo red (CR, concentration of 12 mg/L) before and after filtration through FBN-0.4; the left inset is the molecular structure of CR and the right inset photo shows the feed (F, left) and permeate (P, right) of the CR solution. **b** UV-Vis absorption spectra of an ethanol solution of 5 nm Au nanoparticles before and after filtration through FBN-0.4; the right inset photo shows the feed (F, left) and permeate (P, right) of the Au nanoparticle solution in ethanol. **c** UV-Vis absorption spectra of a methanol solution of Evans blue (EB, concentration of 7 mg/L) before and after filtration through FBN-2; the left inset is the molecular structure of EB and the right inset shows the feed (F, left) and permeate (P, right) of the EB solution in methanol. **d** Separation performance versus harsh treatment of the FBN-0.4 membrane for CR in water

Molecular selectivity is a significant parameter for determining the potential application of filtration membranes. Regularly, dye molecules are selected to detect the filtration performance of a membrane because they are soluble and variable as well as being easy to detect (Supplementary Fig. 12). The concentration of dye molecules in the feed and permeate solutions can be detected using UV-Vis spectroscopy. The FBN-0.4 membrane exhibits a water flux of 1500 L m^−2^ h^−1^ and a high rejection rate of >99% for CR (Fig. 4a). The water flux of FBN-0.4 is approximately 1.5 and 16 times higher than that of MXene (100–400 nm sheets)^16^ and reduced graphene oxide (approximately 2 μm) membranes^33^, respectively. The increased performance is attributed to the small size of the BN flakes and the accessory nanochannels generated in the membranes, as confirmed by the SEM and AFM data. To investigate the potential nanochannels of the FBN-0.4 membrane, more molecules were tested. When the membrane was used for smaller molecules (Supplementary Fig. 13), such as MB, Rhodamine B (RhB), and Rhodamine 6 G (R6G), the rejection rates of the membrane quickly decrease (approximately 50.3%, 58.3%, 83.8% respectively), but the flux increases to over 1800 L m^−2^ h^−1^. For a larger molecule, e.g., 5 nm Au nanoparticles, the membrane can block nearly all of the nanoparticles, showing a >99% rejection rate and good water flux (1230 L m^−2^ h^−1^).

To gain further insight into the permeation and separation performances, FBN membranes with different thicknesses were investigated to balance the water flux and rejection rate^11,48^. As shown in Supplementary Fig. 14a-c when the thickness of the FBN membrane increases with a thickness of 1 μm (FBN-1), the rejection rates improve, showing 94.1% rejection rate for MB, >99% rejection rate for RhB and 98.5% rejection for R6G. During the filtration, when the thickness of the FBN membrane increases, some of the defects along the layers are covered by upper or lower layers. Then, the water flows through the thicker membranes more slowly than thinner ones. However, FBN-1 shows a low rejection rate for K_3_Fe(CN)_6_ (17%) because of the small molecular dimensions (0.9 nm × 0.9 nm) (Supplementary Fig. 14d). Therefore, a thicker FBN membrane has a good separation performance with high flux rate in aqueous media for large dye molecules.

When the CR was dispersed in ethanol, the rejection rate of the FBN-0.4 membrane rapidly decreases to 54% (Supplementary Fig. 15) compared with that in water due to the solvation effect on the membrane. To achieve a high separation in an organic solution, FBN-2 was used for the CR separation in ethanol. Interestingly, FBN-2 showed a high rejection rate of >99% for CR and high flux of 330 L m^−2^ h^−1^ (Supplementary Fig. 16a and c). Moreover, even when the feed concentration of the Au nanoparticles was tripled, the FBN-0.4 membrane still shows a >99% rejection rate (Fig. 4b and Supplementary Fig. 16b, 16d-f). This further demonstrates that the rejection of molecules is dominated by a molecular sieving mechanism. Furthermore, FBN membranes with different thicknesses were used for methylene blue (MB), acid fuchsin (ACF), CR, and EB separations in methanol solutions. FBN-2 can separate the dye molecules well in methanol and shows high rejection rates for MB (60%), ACF (88.7%), CR (>99%) and EB (>99%) depending on the molecular size while maintaining high fluxes (Fig. 4c, Supplementary Fig. 17 and Supplementary Table 3). Then the FBN-8 showed an improved separation rate for the MB, with an improved rejection rate of 93%. The improved rejection performances result from the thicker membrane, which creates more and longer flow routes for more effective sieve solutes^49^. Therefore, the separation performance of the FBN membranes is affected by the thickness of the membrane, the size of the feed molecules, and the solvents.

It is well known that a good separation membrane should have a high performance, such as a high flux and high rejection rate, and it should also be robust, i.e., applicable in basic, acidic or oxidative environments^33^. To investigate the robustness of the membranes, the FBN-0.4 membranes were soaked in 0.5 M H_2_SO_4_, 0.5 M KOH, and 0.5 M HNO_3_ for one month and used for permeation. Notably, the FBN-0.4 membrane maintained a super-high flux of approximately 1550 L m^−2^ h^−1^ and a >99% rejection rate for CR in water after soaking, demonstrating the excellent stability of the FBN membranes, which is attributed to the excellent chemical stability of BN (Fig. 4d and Supplementary Fig. 18). Furthermore, the FBN-2 membrane was also investigated in methanol for EB molecular separation after 30 periodic operations of water filtration. Importantly, the FBN-2 membrane showed a very stable separation performance (rejection: >99%) (Supplementary Fig. 19).

**Fig. 5:**
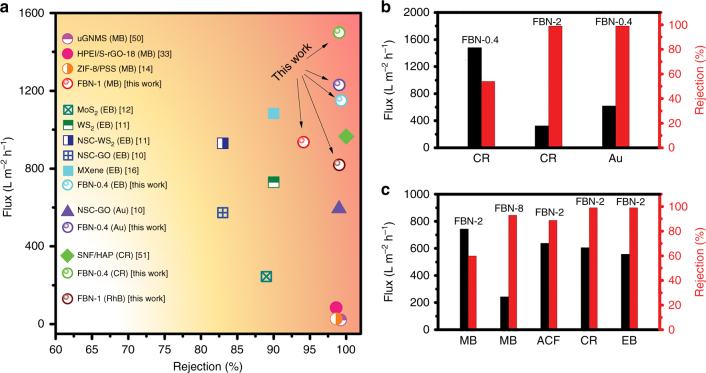
Separation performance of the functionalized boron nitride membranes. **a** Comparison of the separation performance of the FBN membranes versus other reported membranes in aqueous media^9–12,16,33,53,54^. **b** Separation performance of FBN for CR (2.5 nm × 0.7 nm, concentration of 50 mg/L) and Au nanoparticles (5 nm) in ethanol solution. **c** Separation performance of FBN membrane versus molecular size in methanol solution (MB: 1.4 nm × 0.6 nm, concentration of 1.5 mg/L; ACF: 1.2 nm × 1.1 nm, concentration of 3 mg/L; CR: 2.5 nm × 0.7 nm, concentration of 13 mg/L; EB:1.2 nm × 3.1 nm, concentration of 7 mg/L). For details, see the supporting information (Supplementary Tables 1–5)^33,55–57^. The flux values refer to the solvent filtration rate from the solutions

Therefore, compared with those of other membranes composed of other 2D nanomaterials, including graphene derivatives, exfoliated dichalcogenides, and MXene, the FBN membranes have excellent separation and fluxes in aqueous media under similar conditions (Fig. 5a, Supplementary Tables 1 and 4). For example, FBN-0.4, with a thickness of 400 nm, has a super flux of 1230 and 1500 L m^−2^ h^−1^ bar^−1^ and a high rejection rate of >99% for 5 nm Au and CR, respectively. When increasing the FBN thickness to 1 µm (FBN-1), the membrane with a thickness of 1000 nm also exhibits fluxes of 940 and 820 L m^−2^ h^−1^ bar^−1^ for MB (94.1% rejection rate) and RhB (>99% rejection rate), respectively. In organic solvents (Fig. 5b, c, Supplementary Tables 2, 3 and 5), the thicker FBN membranes exhibited good separation and permeance performances. For example, FBN-2, with a thickness of 2 μm, has a >99% rejection for CR in ethanol with a high flux of 330 L m^−2^ h^−1^, and shows increased rejection rates for MB (60%), ACF (88.7%), CR (>99%) and EB (>99%) in methanol depending on the molecular size. Thus, all the results indicate that the FBN membranes are promising for practical industrial applications.

There are four aspects contributing to the excellent flux and separation performance of FBN membranes. First, the superhydrophilic property of FBN membrane benefits to the high permeance^10–12,16^. The contact angle of the FBN-2 membrane was measured. As shown in Supplementary Fig. 20, the membrane exhibits a contact angle of about 40° indicating the superhydrophilic property of FBN membrane. Second, the FBN membrane contains small FBN flakes (100–300 nm, Supplementary Fig. 8) and can generate more nanochannels between the FBN flakes for small molecules to pass through. Similar to the reports of other 2D nanomaterials^10–12,43^, the stacked FBN flakes have more transport routes, facilitating the transport speed in the membrane. The nanochannels created by the FBN membrane fabrication process are the main reason for the increased permeation performance. When the thickness of the FBN membrane increases, the solutes must pass through longer routes, decreasing the corresponding flux but increasing the separation capability of the membrane. Third, the spaces between the FBN flakes are stable, supporting the confirmed nanochannels in the lamellar membrane. Similar to the study by Qin et al.^26^, a small-angle X-ray scattering (SAXS) analysis was performed on the FBN membrane (Supplementary Fig. 21). The results demonstrate that there are many nanochannels between the FBN flakes and small molecules can pass through these nanochannels. The actual spaces (nanochannels) between the FBN flakes were calculated to be within 8–18.3 Å (See Supplementary Fig. 21 for details), which are wide enough for 2–5 layers of water molecules between the FBN flakes. Thus, a membrane containing BN flakes shows obvious fluidic phenomena^31^. Finally, the low pressure (1 bar) and the fast stirring (800 rpm) can induce a very little concentration polarization, resulting in a better permeance and separation permeance (Eq. (1)–(4) in Supplementary Note 1)^50,51^. Therefore, the distinct nanochannels, sufficient nanochannels and lamellar microstructures benefit to the high water permeance rate and good separation performance of the membrane in aqueous and organic solvents. Furthermore, the current FBN membrane has a negative surface charge (−33 mV), which has little effect on the separation performance. Therefore, the membrane can be modified by different surface charges (positive or negative functional groups) to improve the separation of different charged solutes and to enhance the anti-fouling ability^52^.

In summary, we developed a membrane with an adjustable thickness by filtering highly water-dispersible FBN flakes. The membrane shows water and organic solution resistances and acidic, alkaline, and oxidative media stability. Furthermore, the FBN-based membrane features a high flux and good selectivity in both aqueous and organic solvents due to the small size of the FBN flakes and the stable nanochannels between the FBN flakes. Therefore, these highly efficient FBN membranes are promising for industrial separation and purification applications.

## Methods

### Preparation of the functionalized boron nitride dispersion

The FBN dispersion was prepared using commercial h-BN powder (from Momentive Performance Materials Inc.) and ball milling the powder with urea (from Sigma-Aldrich), which was the functional agent^32^. Typically, the h-BN powder (0.5 g) was mixed with urea (10 g) in a ball milling cell. Then, the mixture was washed with DI water for one week after balling milling it at 400 rpm for 20 h at room temperature. After centrifuging the mixture for 10 min at 3000 rpm, we obtained FBN dispersions in water with a controllable concentration. After calibration of the dispersion, the milk h-BN solution was prepared with a concentration of 3.4 mg/mL.

### Fabrication of the functionalized boron nitride membranes

The FBN membranes were prepared by vacuum filtration of diluted FBN dispersions on nylon microfilter membranes (47 mm in diameter, 0.2 μm pore size). The thickness of the FBN membranes was tailored by changing the added FBN amount. Therefore, the resulting membranes are denoted FBN-*X*, where *X* = 0.4, 0.7, 1, 1.5, 2, 8, and is the thickness of the FBN membrane (μm). Once the filtration is complete, the produced membrane was allowed to sit for 30 min before further transform and test.

### Separation performances of the functionalized boron nitride membranes

All the collected data are averages of three parallel tests. Parallel filtration experiments were performed using a positive pressure setup (Supplementary Fig. 22). The working area of the h-BN membrane is 7.07 cm^2^ (an effective round area with a diameter of 3 cm). A compressed nitrogen bottle was used and the applied pressure was fixed at 1 bar. Every 40 mL filtered solvent was collected with a sealed container. The dye separation tests were also performed using the same setup. Feed volume was 80 mL. The concentrations of the feed, permeate, and retentate solutions were measured by using a UV-Vis spectrophotometer after collecting 40 mL of the solvents through the membranes. The pure solvent permeance (*F*) was calculated using

1 $$F = V/(A \times t \times P)$$

where *V* is the volume of the collected solvents, *A* is the effective membrane working area (7.07 × 10^−4^ m^2^), *t* is the permeation time (h), and *P* is the fixed applied pressure of 1 bar. The rejection (*R*) rate of the markers was calculated using

2 $$R = \left( {1 -C_{\mathrm{P}}{C_{\mathrm{R}}}} \right) \times 100\%$$

where *C*_P_ and *C*_R_ are the concentrations of markers in the permeance and retention solutions. These data came from the UV-Vis spectra. Eq. (1) can only be used for pure solvents.

### Activation energy (*E*_a_) calculation

The Arrhenius equation,

3 $$k = Ae^{( - E_{\mathrm{a}}/RT)}$$

where *k* is the solvent permeation speed (L m^−2^ h^−1^), *A* is the pre-exponential factor (L m^−2^ h^−1^), *E*_a_ is the activation energy associated with the permeation process (kJ mol^−1^), *R* is the gas constant (kJ mol^−1^ K^−1^), and *T* is absolute temperature (K). By taking the log of both sides of Eq. (3) and using *R* (8.314 × 10^−3^ kJ mol^−1^ K^−1^), the *E*_a_ can be evaluated from the ln (*k*) vs. 1000/T plot.

### Soaking test of the functionalized boron nitride membranes

The prepared membranes were soaked in 0.5 M H_2_SO_4_, 0.5 M NaOH, and 0.5 M HNO_3_, respectively, for one month. Then, samples were carefully removed for further experiments.

### Characterization

The XRD measurements were performed on a Panalytical X’Pert PRO apparatus with Cu K_α_ radiation. Small-angle X-ray scattering (SAXS) was performed on the SAXS/WAXS beamline at the Australian Synchrotron (AS). The Pilatus 1 M detector was used for data collection. The 0.6 m camera length was selected to give a *q*-range of 0.05–1.6 Å^−1^. The X-ray beam with wavelength *λ* = 0.62 Å (20 keV) and a size of 250 μm horizontal x 150 μm vertical (fwhm), was selected to prevent damage from long-time exposure. The exposure time was 2 s. Empty measurements were subtracted from the final data. The data were reduced using Scattering Brain software developed at AS and TIT2D software. The SEM analysis was performed on a Zeiss Supra 55 VP. The membrane was first sputtered with a 2 nm carbon coating. The AFM measurements were performed on a Cypher atomic force microscope. The FTIR and UV-Vis spectra were recorded using a Nicolet 7199 FTIR spectrometer and a Cary 3 UV-Vis spectrophotometer, respectively. The Raman spectra were collected using a Ranishaw Raman spectrometer with a wavelength of 633 nm. The contact angle was tested with a contact angle goniometer (CAM101, KSV) and the data was an average of 6 parallel measurements.

### Data availability

The data that support the findings of this study are available from the corresponding author on reasonable request.

## Electronic supplementary material

Supplementary Information
