# Supplementary material for: Functionalized boron nitride membranes with ultrafast solvent transport performance for molecular separation
Source: Nat Commun. 2018 May 15;9:1902. doi: 10.1038/s41467-018-04294-6 (PMC5954095; doi:10.1038/s41467-018-04294-6)
Supplement: Supplementary file 1 — Supplementary Information [file 41467_2018_4294_MOESM1_ESM.pdf]

Supplementary Information

Functionalized boron nitride membranes with ultrafast solvent transport performance for molecular separation

Chen et al.

Supplementary Figures

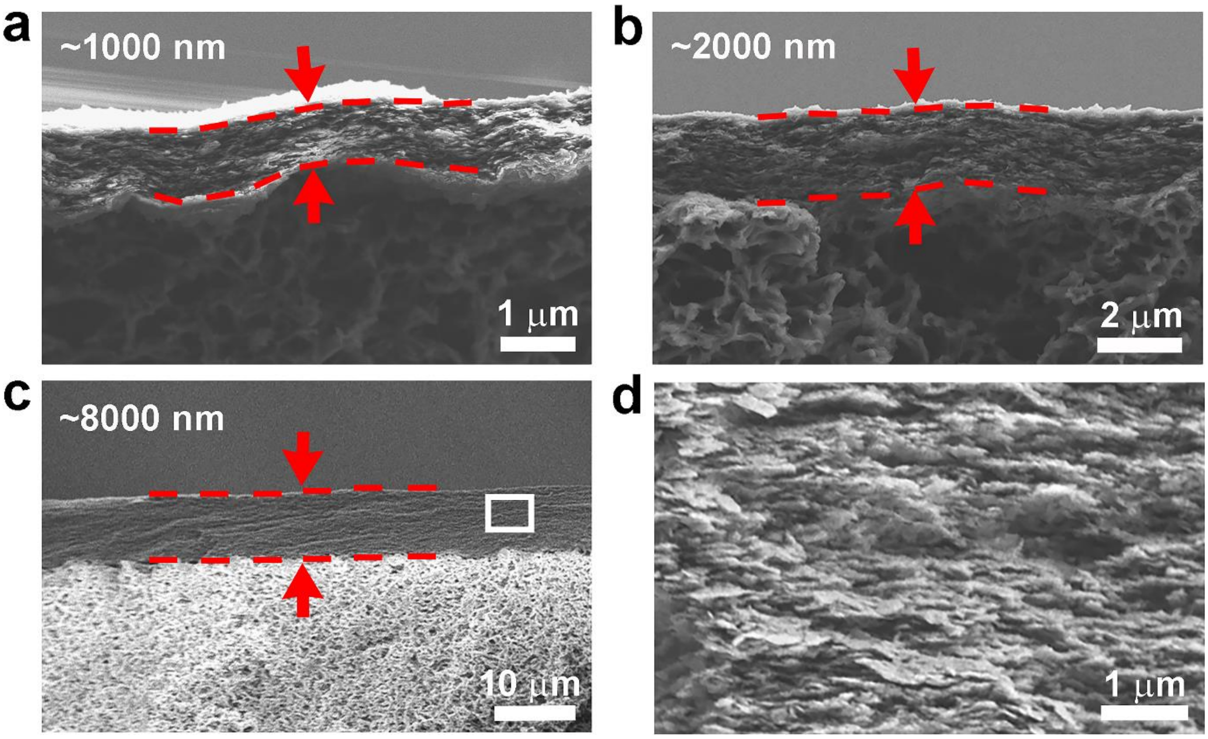

Supplementary Figure 1. The cross-sectional SEM images of (a) FBN-1, (b) FBN-2, (c) FBN-8, and (d) an enlarged image highlighted in the square in (c).

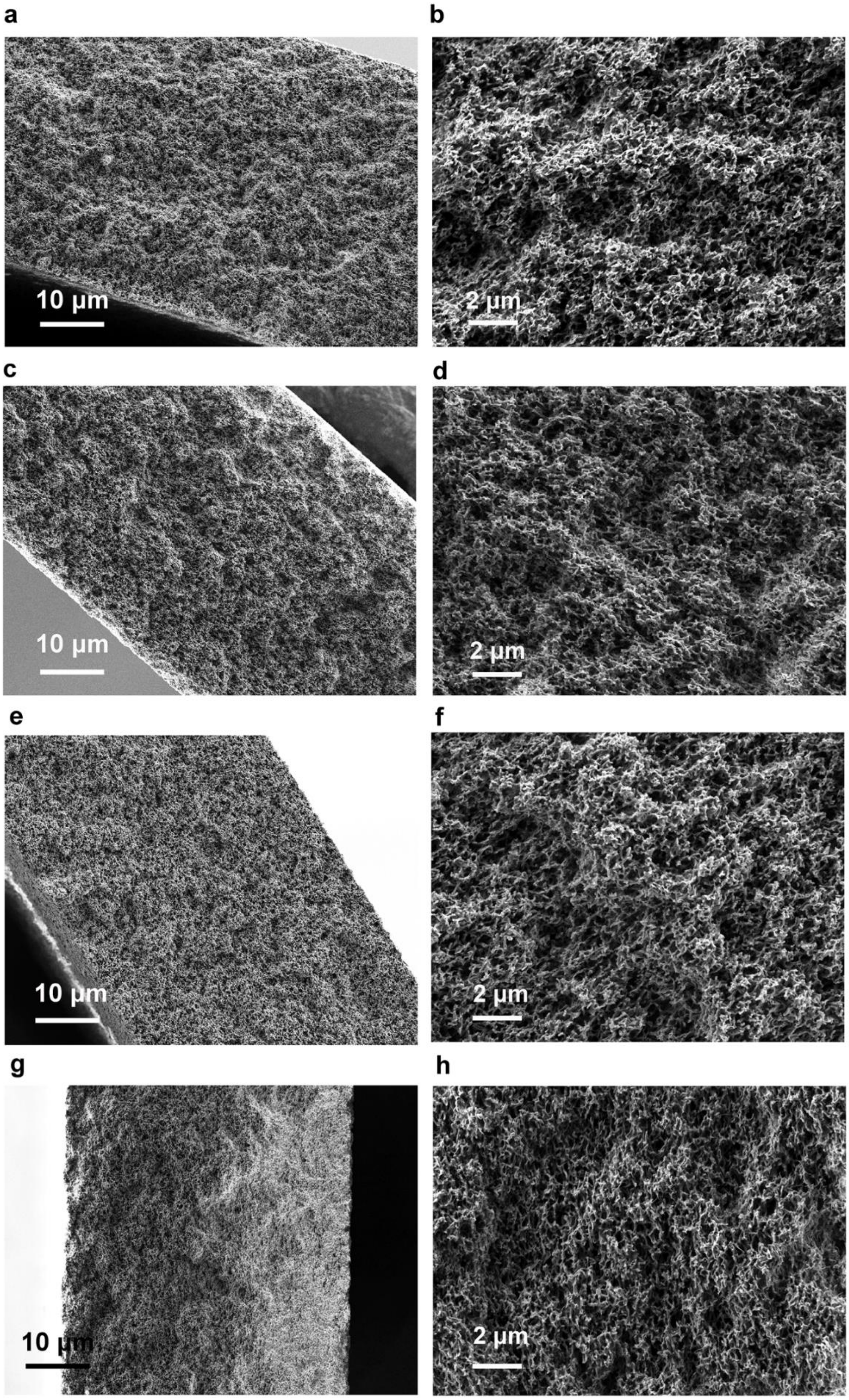

Supplementary Figure 2. SEM images of the nylon membranes after one month of soaking in H_2SO_4 (a, b), NaOH (c, d), HNO_3 (e, f) and the pristine nylon membrane (g, h).

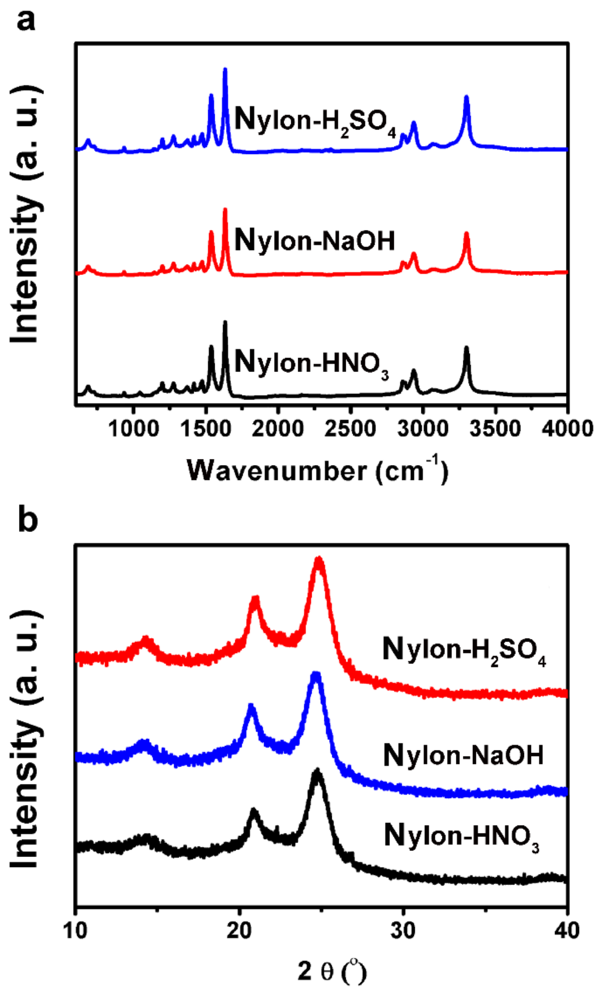

Supplementary Figure 3. FTIR-ATR (a) and XRD (b) spectra of the nylon membranes after soaking in H_2SO_4, NaOH, and HNO_3.

Supplementary Figures 2 and 3 were used to confirm that the nylon membrane was chemically and physically stable after one month of harsh treating conditions. It should be noted that the treated nylon membrane showed a weak mechanical strength, and care should be taken to transfer it to the setup for further tests.

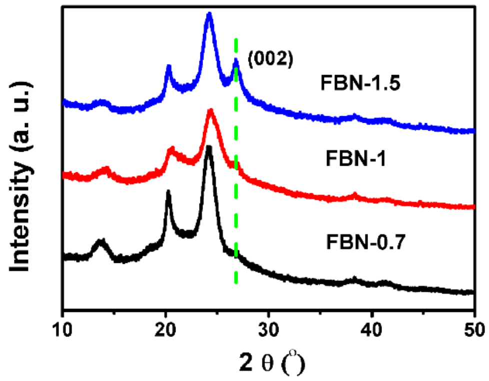

Supplementary Figure 4. XRD patterns of FBN-X with X = 0.7, 1, and 1.5 μm of the FBN membranes.

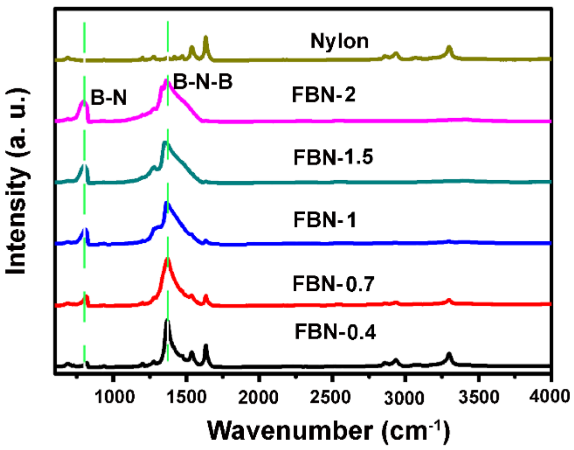

Supplementary Figure 5. FTIR-ATR spectra of different thickness of FBN on nylon.

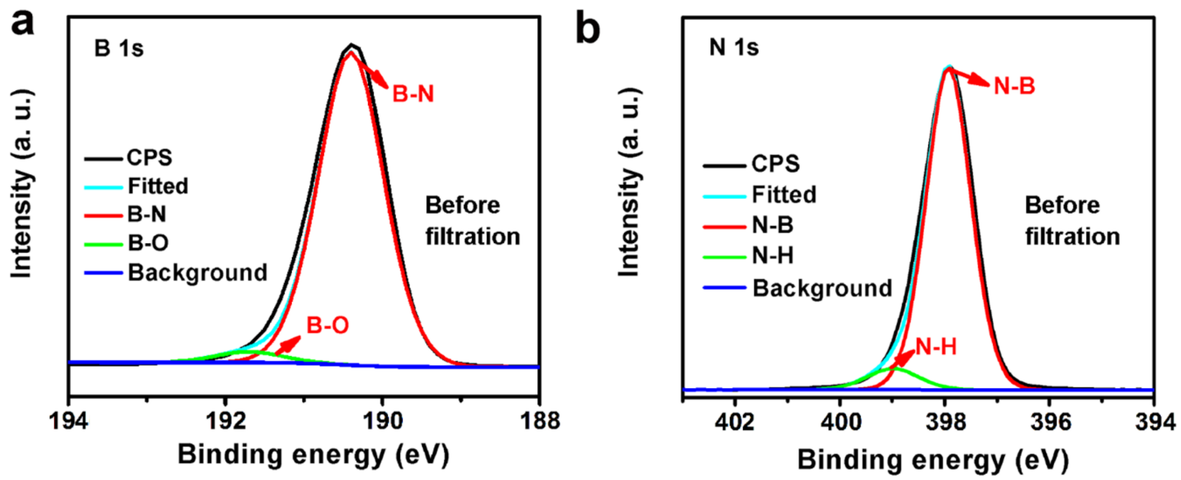

Supplementary Figure 6. XPS spectra of FBN membrane. (a) B 1s, (b) N 1s.

As shown in Supplementary Figure 6, the high-resolution B 1s and N 1s X-ray photoelectron spectroscopy (XPS) results directly confirm that the amino function groups are well connected in the FBN, which is helpful for change the hydrophobicity into hydrophilicity of FBN.

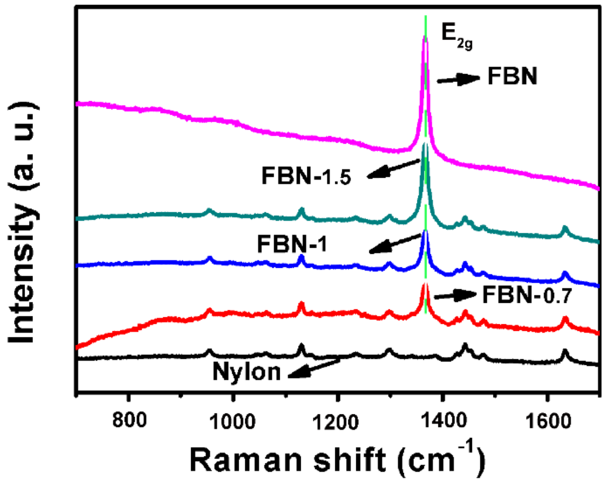

Supplementary Figure 7. Raman spectra of different thickness of FBN on nylon membrane.

As shown in Supplementary Figures 4, 5 and 7, the characteristic peaks of BN linearly increase with the thickness of the FBN membrane.

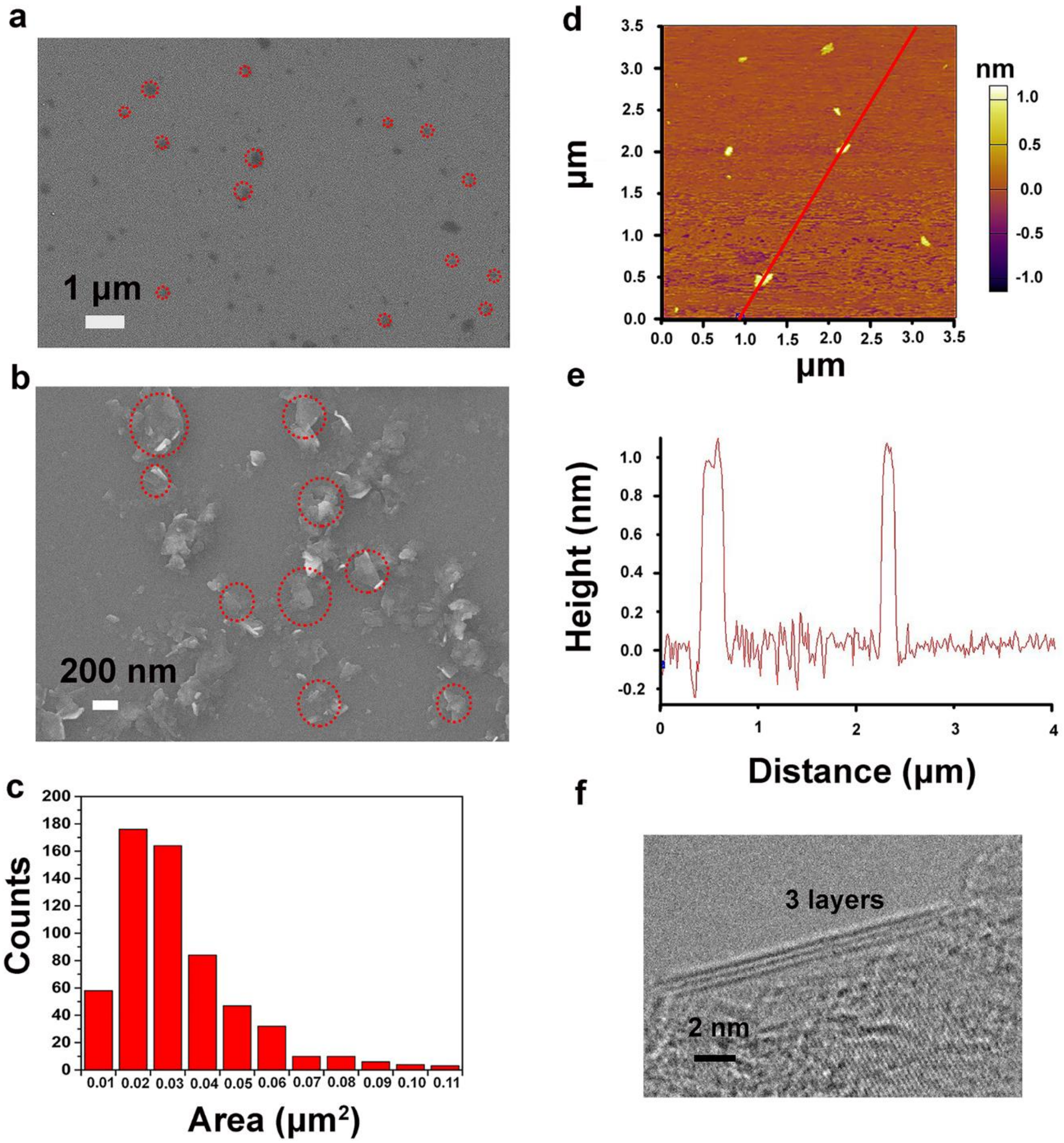

Supplementary Figure 8. (a) A typical SEM image of the FBN flakes on a polished p-type silicon substrate. (b) A typical SEM image of the FBN flakes on a normal Si substrate. (c) A statistical distribution of areas of the individual sheets based on the measurements of 592 FBN flakes. (d) A low-magnification AFM image of the FBN. (e) A height profile of the FBN flakes from (d). (e) HRTEM image of the edge folding of sheet with three FBN layers.

Supplementary Figures 8a and b show a typical SEM image of the FBN flakes on a polished p-type silicon and normal Si substrate, respectively. The polished p-type silicon wafer pieces allowed for imaging the electrically insulating the FBN flakes with good contrast. The FBN flakes were diluted in water and dropped onto the Si and dried for imaging. The FBN was

circled with red dashed circles on a normal substrate. The area of the FBN flakes was calculated using ImageJ (available from the NIH as a freeware). Those flakes which are overlapped were ‘ruled out’ during the image analysis. Supplementary Figure 8c shows a statistical distribution of the size based on 592 FBN flakes. It can be seen that most FBN flakes have an area of 0.01~0.06 μm^2. It means that the lateral FBN flakes dimension ranging from 100 nm to 300 nm. Supplementary Figure 8d, e and f further confirm that the FBN flakes are constituted with ~2-4 monolayers, with sizes distribution from 100 nm to 300 nm, which is in agreement with the SEM data.

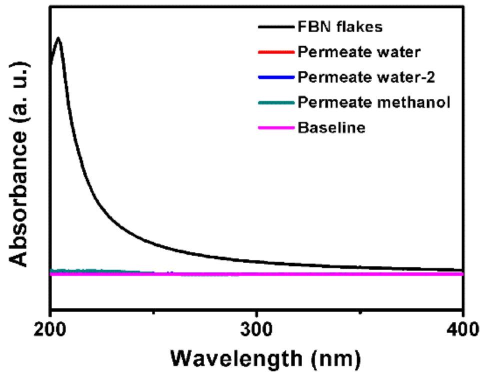

Supplementary Figure 9. The comparison between the UV-Vis absorption spectra of 200 μL pure FBN dispersion in 50 mL of DI water (FBN flakes, black line) and the permeate after the first cycle for water (Permeate water, red line), the permeate after second cycle for water (Permeate water-2, blue line), the permeate after first cycle for methanol (Permeate methanol, dark cyan line) through FBN-2 membrane and the baseline (baseline, magenta line).

The UV-Vis absorption spectra of the pure FBN flakes in a water solution, the permeate water after the first and second filtration, and the permeate methanol after the first filtration through the FBN-2 membrane are shown in Supplementary Figure 9. It is hard to see the FBN peak in the permeate water and methanol after filtration by the FBN membrane compared with that of the pure FBN solution, indicating almost no leaching of BN into the solution.

Here, to avoid the leakage of FBN into solution, three points should be noted. First, before filtering a FBN solution to create a FBN membrane, 20 mL of water should be poured onto the nylon membrane to wet the nylon membrane and help the FBN flakes stack well in the filtration assembling process. After the filtration of FBN flakes on the nylon, the FBN membrane was carefully transformed in a stainless steel dead-end filtration device used for the solvent filtration experiments. Second, the experimental solvents should be slowly poured along the inner wall of the device to reach the target volume (80 mL) and avoid breaking the FBN membrane. Third, the applied gas pressure can be gradually increased to 1 bar and the solvent should be left about 40 mL every time.

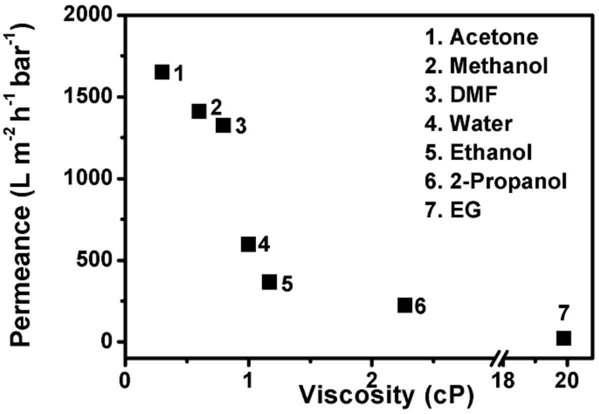

Supplementary Figure 10. The permeation of different pure solvents through FBN-2 membrane.

All the FBN membranes were used for pure solvent filtration under the same condition used for FBN-2.

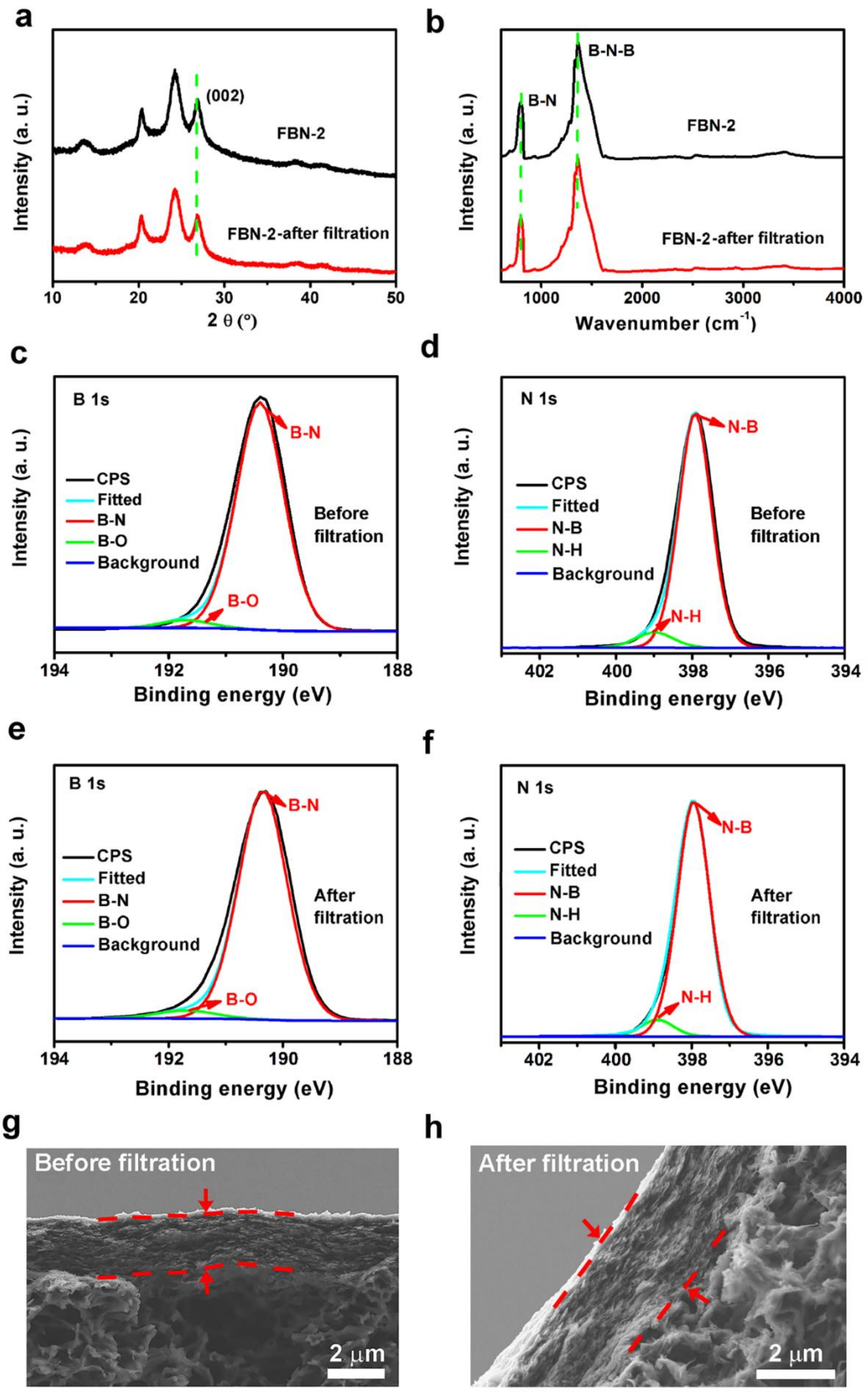

Supplementary Figure 11. The stability of the FBN membrane. The XRD (a), FTIR-ATR (b), XPS (c, d, e, f), and the cross-sectional SEM images (g, h) of FBN-2 before and after filtration.

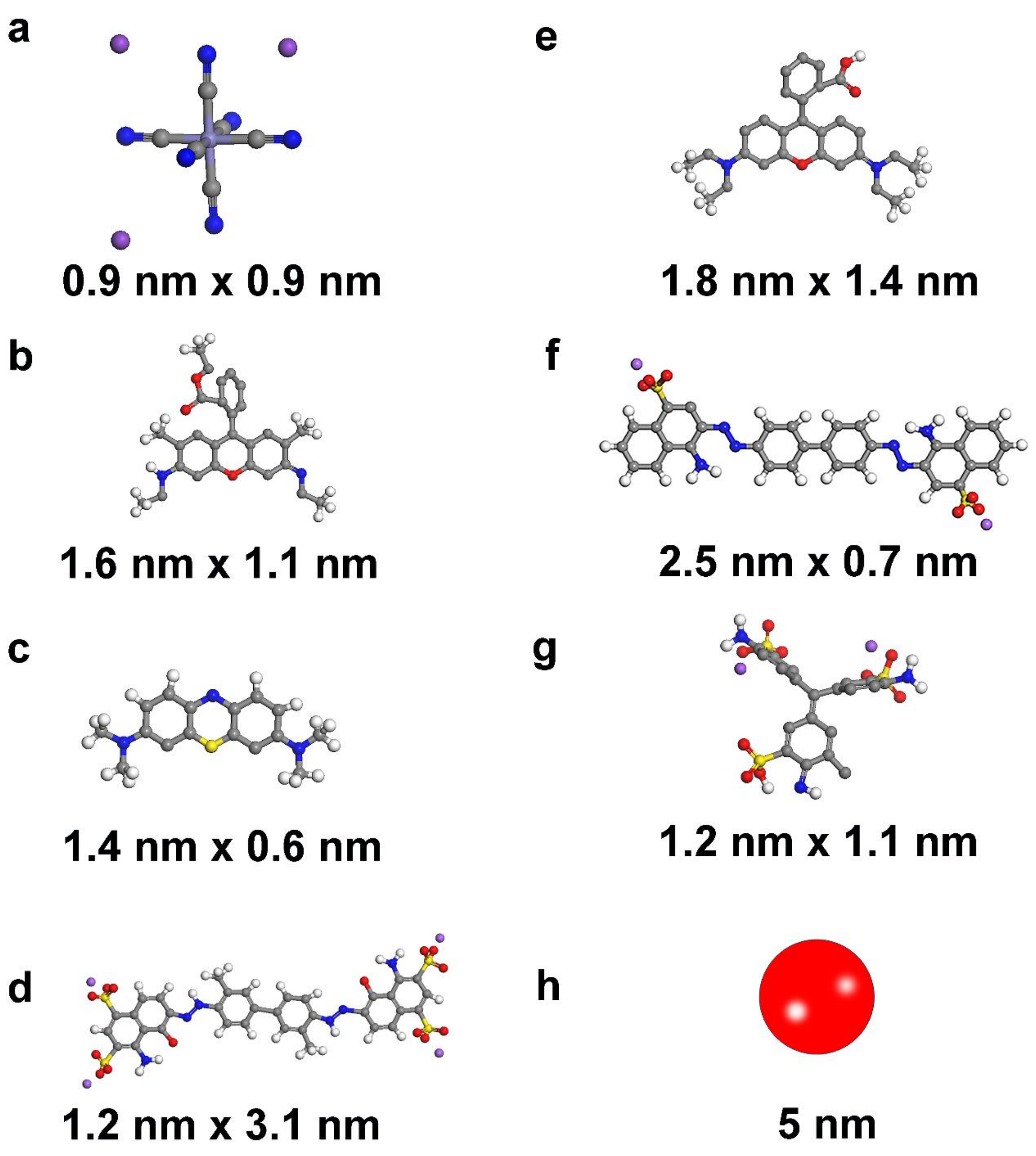

Supplementary Figure 12. The chemical structures of the dyes used for the molecular separation experiments, (a) $K_3Fe(CN)_6$, (b) R6G, (c) Methylene blue, (d) Evans blue, (e) RhB, (f) Congo red, (g) Acid fuchsin and (h) Au nanoparticles.

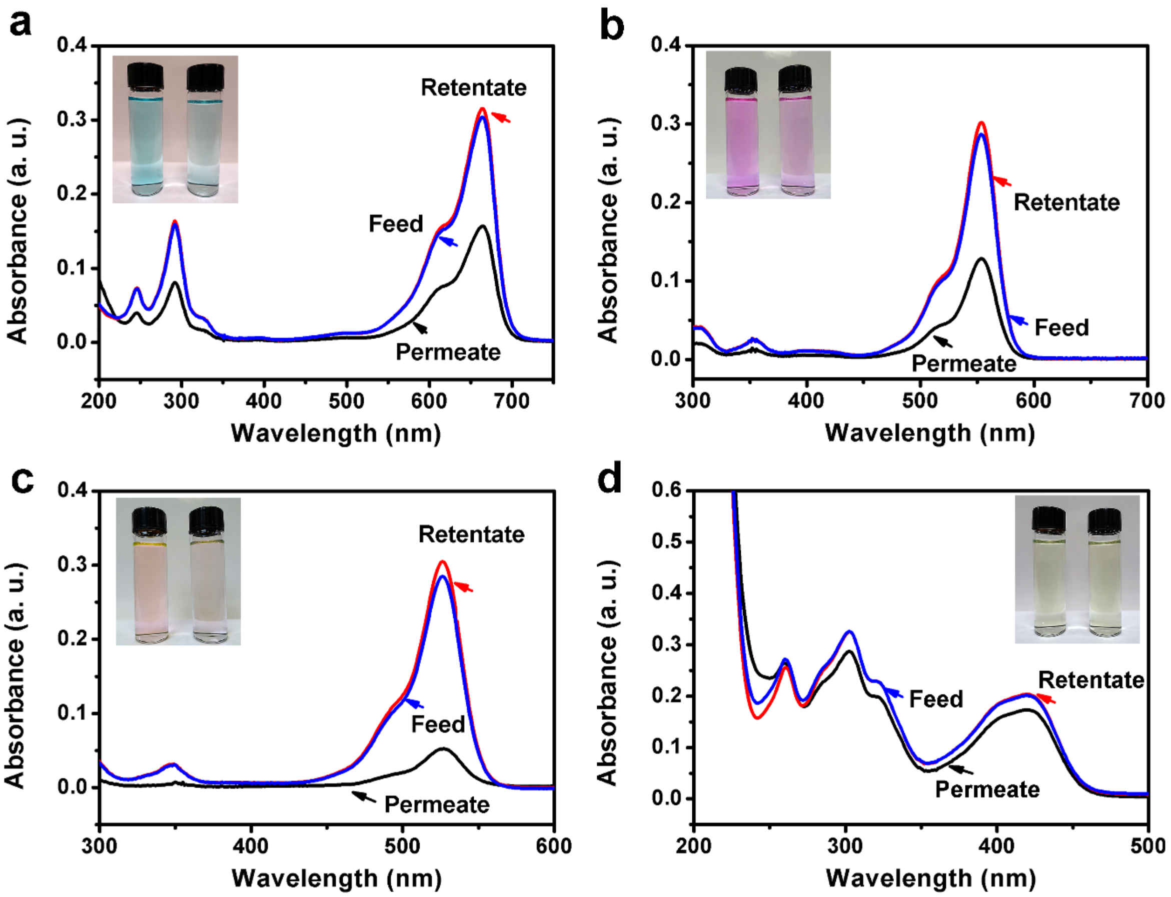

Supplementary Figure 13. UV-Vis absorption spectra of MB (a), RhB (b), R6G (c) and $K_3Fe(CN)_6$ (d) in water before and after filtration through a FBN-0.4 membrane.

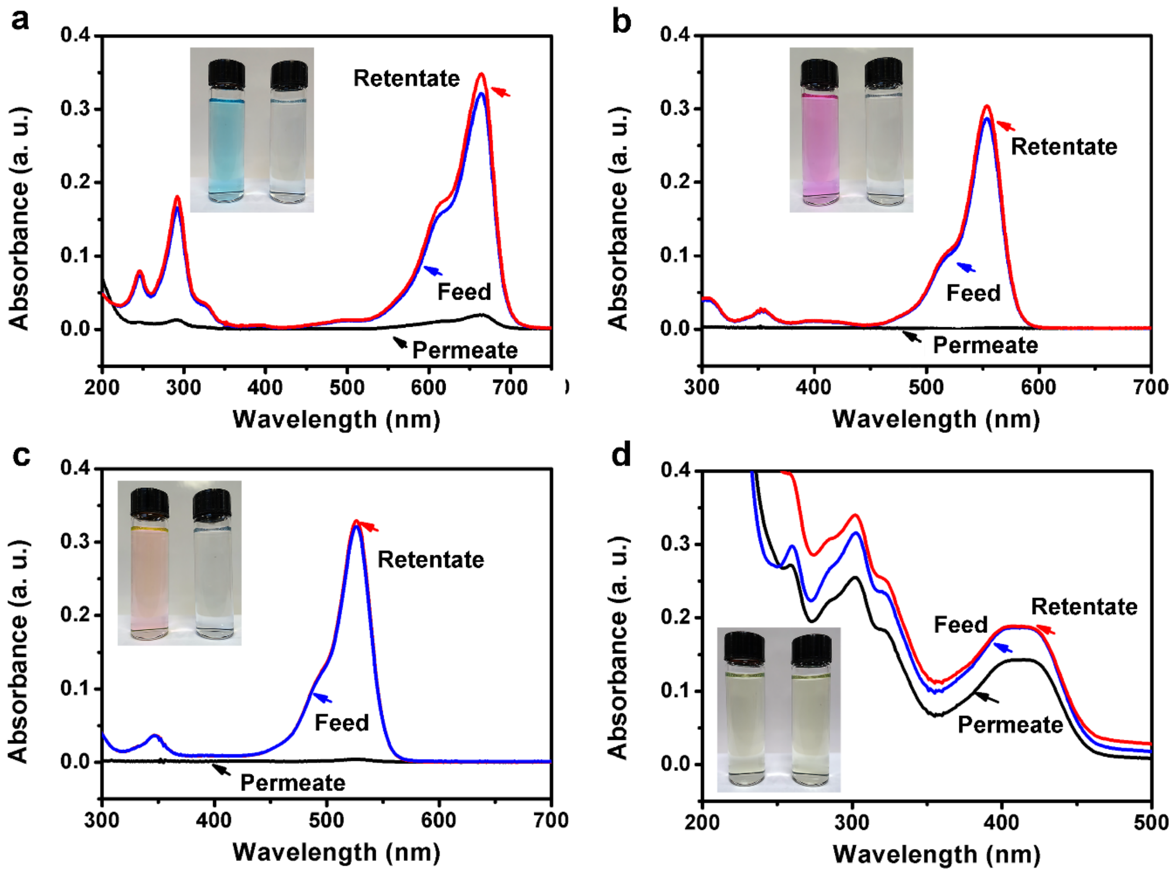

Supplementary Figure 14. UV-Vis absorption spectra of MB (a), RhB (b), R6G (c) and $K_3Fe(CN)_6$ (d) in water before and after filtration through a FBN-1 membrane.

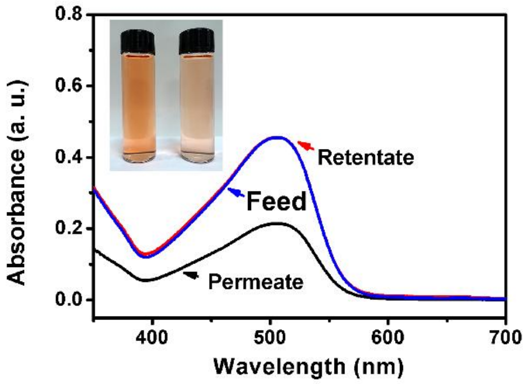

Supplementary Figure 15. UV-Vis absorption spectra of Congo red in ethanol before and after filtration through a FBN-0.4 membrane.

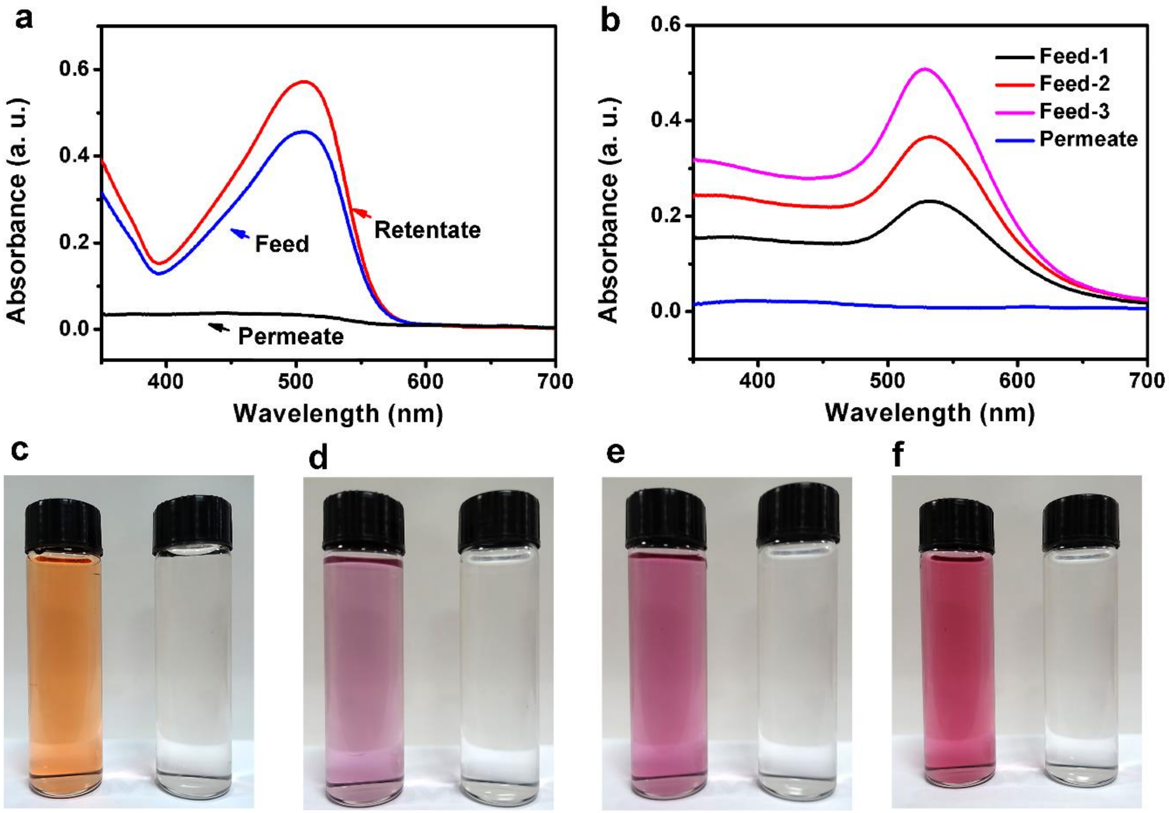

Supplementary Figure 16. UV-Vis absorption spectra of Congo red through FBN-2 membrane (a) and Au nanoparticles through FBN-0.4 membranes (b) in ethanol before and after filtration, respectively. (c) The feed and permeate solution of CR in ethanol after the FBN-2 membrane separation. (d) (e) (f) The increased concentration of Au in ethanol in the feed and permeate solutions after the FBN-0.4 membrane separation.

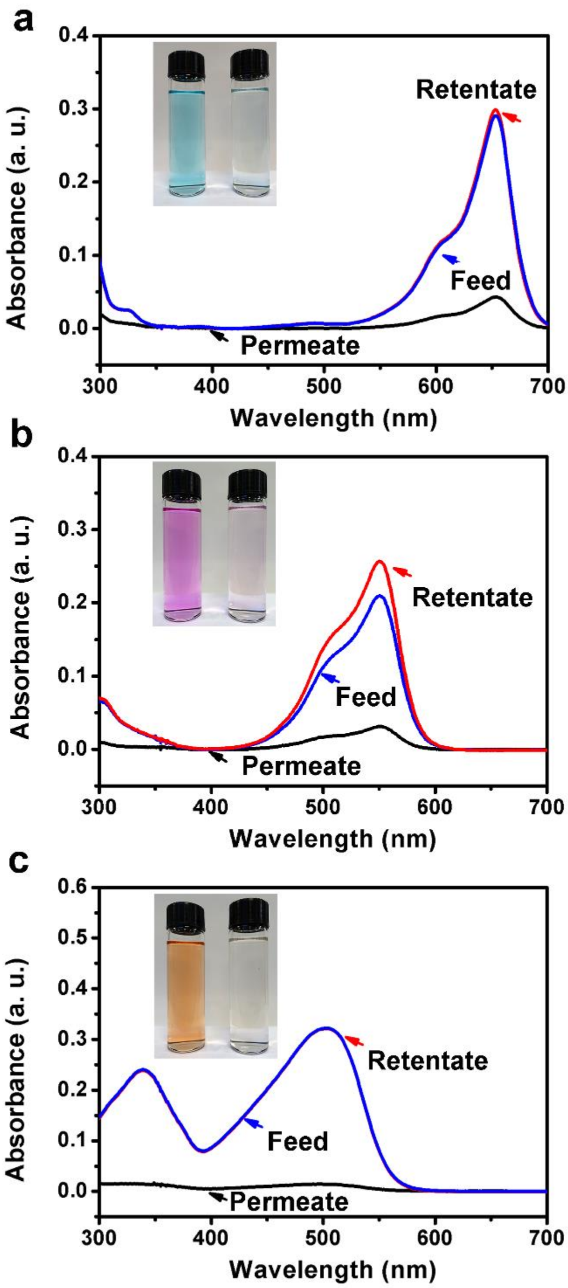

Supplementary Figure 17. UV-Vis absorption spectra of small MB molecules in methanol before and after filtration through FBN-8 membranes (a), ACF (b) and Congo red (c) in methanol before and after filtration through FBN-2 membranes.

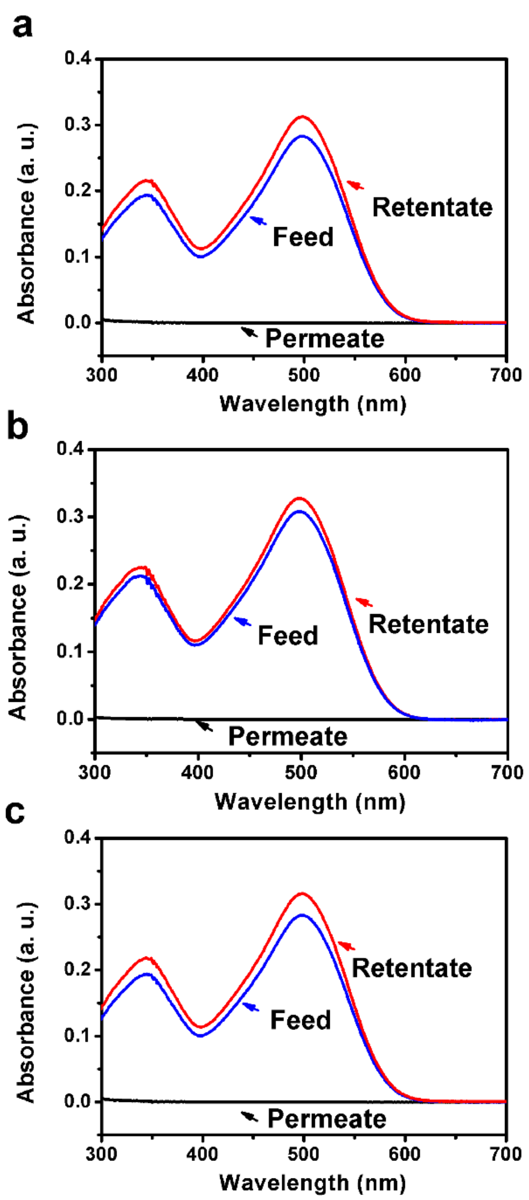

Supplementary Figure 18. UV-Vis absorption spectra of CR in water before and after filtration through a FBN-0.4 membrane after one month soaking in 0.5 M H_2SO_4 (a), 0.5 M NaOH (b) and 0.5 M HNO_3 (c), respectively.

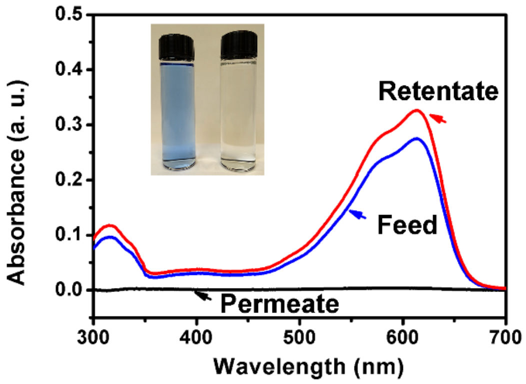

Supplementary Figure 19. UV-Vis absorption spectra of EB in methanol before and after filtration through a FBN-2 membrane after 30 periodic operations of water filtration.

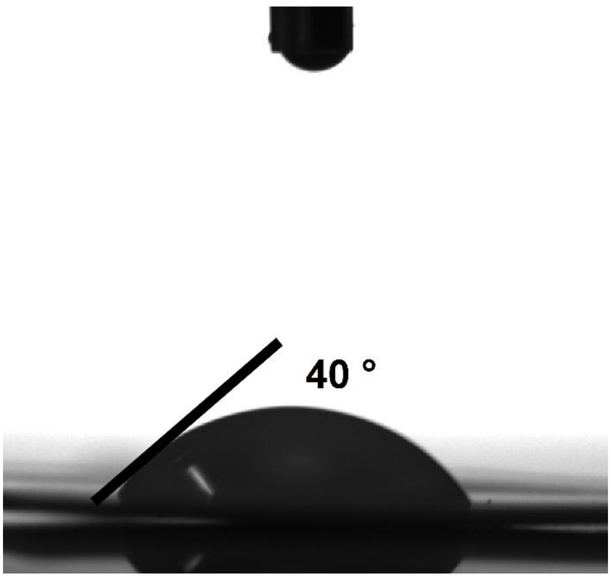

Supplementary Figure 20. The contact angle of FBN-2. The contact angle is about 40 ° on an average of 6 measurements with a contact angle goniometer (CAM101, KSV).

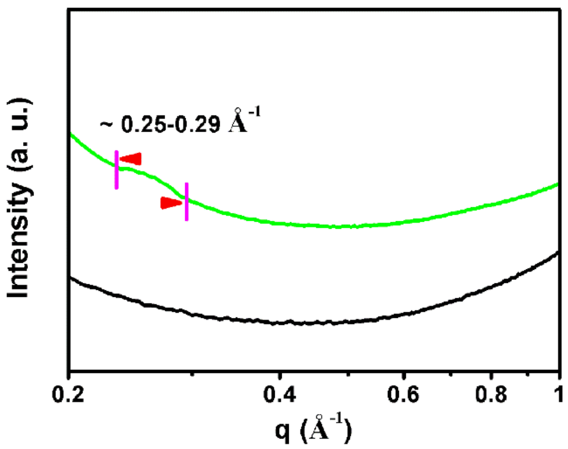

Supplementary Figure 21. SAXS of the FBN membrane. The green line is from the fully hydrated FBN membrane, and the black line is from the dry FBN membrane.

Supplementary Figure 21 shows the SAXS patterns from dry and fully hydrated BN-based membranes. The SAXS results show a wide peak in the range of 0.25-0.29 $Å^{-1}$ and centered at 0.27 $Å^{-1}$. Using Bragg's law ($d = 2\pi/q$), there should be a gap of 21.65~25.12 Å. As confirmed in Figure 2d and Supplementary Figure 8, the FBN flakes should be 2-4 layers. That means there is an actual gap of 8-18.3 Å between the sheets. Therefore, there should be a space (nanochannel) of 8-18.3 Å in the FBN flakes after reducing the thickness of the FBN, which can explain why small molecules can pass through the FBN membrane easily and large nanoparticles (Au, 5 nm) are blocked.

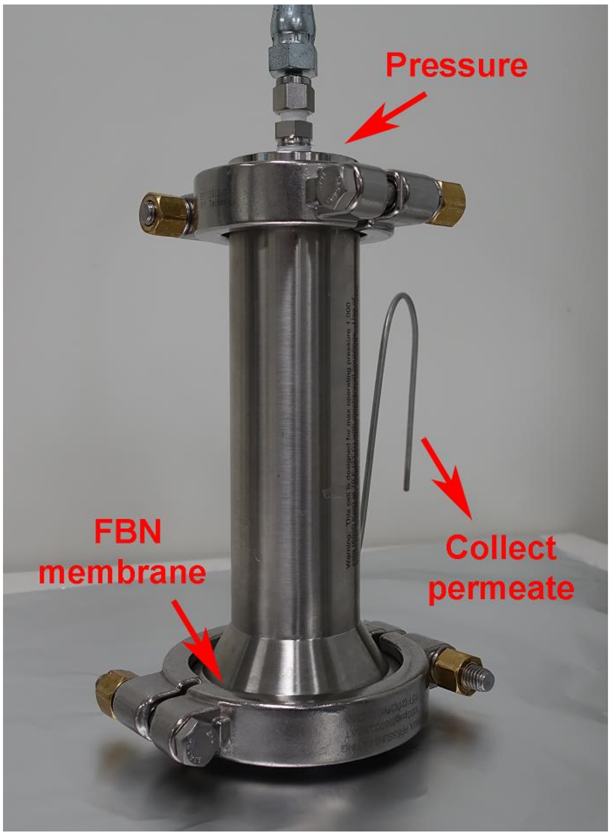

Supplementary Figure 22. Photograph of the dead-end filtration device equipped with a compressed nitrogen bottle, which could be fixed the pressure of 1 bar.

A commercial dead-end filtration device was applied for solvent permeation and molecular separation, as shown in Supplementary Figure 22. For experiment, much care should be taken when transform the FBN membrane on the bottom of the device. Then the solvent was poured into the device along the wall slowly. The first 10 mL volume of the solvent should not be timed for permeation rate calculation, which was a must to make the permeation speed stable. After collecting the target volume, for solvents permeation rate calculation, the whole volume should reduce 10 mL, while for the separation experiment, the first 10 mL solvent should be added into the collected solvent to test the concentration of the permeate.

Supplementary Table 1. Comparison of the performance of the FBN-0.4 and FBN-1 membranes for the separation of different molecules with different sizes in water.

Dye	Solvent	Molecular size / nm^2	Concentration	0.4 µm		1 µm	
				Flux.[a]	Rej. / % [b]	Flux.	Rej. / %
Evans blue (EB)	water	1.2 x 3.1	4 mg/L	1150	> 99	/	/
CR[c]		2.5 x 0.7	12 mg/L	1500	> 99	/	/
MB[d]		1.4 x 0.6	2 mg/L	1860	50.3	940	94.1
RhB		1.8 x 1.4	1.5 mg/L	2050	58.3	820	> 99
R6G		1.6 x 1.1	2 mg/L	2105	83.8	810	98.5
$K_3Fe(CN)_6$		0.9 x 0.9	60 mg/L	2200	15	950	17
Au nanoparticles		5.0 x 5.0	N/A	1230	> 99	/	/

[a] Flux: the unit is L $m^{-2}h^{-1}$, [b] Rej: rejection, [c] CR: Congo red, [d] MB: Methylene blue.

Supplementary Table 2. Comparison of the performance of the FBN-0.4 and FBN-2 membranes for the separation of different molecules with different sizes in ethanol solution.

Dye	Solvent	Molecular size / nm^2	Concentration	0.4 μm		2 μm	
				Flux. [a]	Rej. / % [b]	Flux.	Rej. / %
CR[c]	ethanol	2.5 x 0.7	50 mg/L	1480	54	330	>99
Au nano particles		5.0 x 5.0	N/A	620	>99	/	/

[a] Flux: the unit is L m^{-2} h^{-1}, [b] Rej: rejection, [c] Congo red

Supplementary Table 3. Comparison of the performance of the FBN-2 and FBN-8 membranes for the separation of different molecules with different sizes in methanol solution.

Dye	Solvent	Molecular size / nm^2	Concentration	2 μm		8 μm	
				Flux.[a]	Rej. / % [b]	Flux.	Rej. / %
MB[c]	methanol	1.4 x 0.6	1.5 mg/L	740	60	240	93
ACF[d]		1.1 x 1.1	3 mg/L	640	88.7	/	/
CR[e]		2.5 x 0.7	13 mg/L	600	> 99	/	/
EB[f]		1.2 x 3.1	7 mg/L	560	> 99	/	/

[a] Flux: the unit is L m^{-2} h^{-1}, [b] Rej: rejection, [c] MB: Methylene blue, [d] ACF: Acid fuchsin, (e) CR: Congo red, [f] Evans blue.

Supplementary Table 4. Comparison of the filtration performance of various membranes in water.

**Membrane**	**Thickness (um)**	**Probe molecule**	**Flux.[a]**	**Rej. / %[b]**	**Reference**
uGNMs	0.022	MB[c]	21.8	99.2	1
HPEI/S-rGO-18	0.018	MB[d]	85.4	98.6	2
MoS_2	1.8	EB[e]	245	89	3
WS_2	0.3	EB[e]	730	90	4
Nanostrands-chenneled WS_2	0.5	EB[e]	930	83	4
ZIF-8/PSS	/	MB[c]	26.5	98.6	5
Nanostrand-channeled GO	2	EB[e]	573	83	6
Nanostrand-channeled GO	2	Au	593	100	6
SNF/HAP	7	CR[f]	964.8	100	7
SWCNT-intercalated GO	0.04	Cytochrome C	700	98.3	8
MXene	0.4	EB[e]	1084	90	9
FBN	0.4	Au	1230	> 99	This work
FBN	0.4	EB	1150	> 99	
FBN	0.4	CR[f]	1500	> 99	
FBN	1	RhB	820	> 99	
FBN	1	MB[d]	940	94.1	

[a] Flux: the unit is L m^{-2} h^{-1}, [b] Rej: rejection, [c] MB: Methyl blue, [d] MB: Methylene blue, [e] EB: Evans blue, [f] Congo red. All flux data were normalized with 1 bar.

Supplementary Table 5. Comparison of the filtration performance of various membranes in organic solvents.

**Membrane**	**Thickness (um)**	**Probe molecule**	**Flux.**[a]	**Rej. / %**[b]	**Reference**
S-rGO-18	0.018	ACF[c]	< 78 (M)	70.1	2
HPEI/S-rGO-18	0.018	MB[d]	72.5 (M)	90	2
MPD-3%-1 min-ACT	0.095	ACF[c]	51.84 (M)	99.9	10
acetylene membrane	0.01	Au (5 nm)	363.8 (E)	100	11
PBI/HPEI	1.5	MB[d]	2.6	>99	12
FBN	0.4	Au (5 nm)	620 (E)	> 99	This work
FBN	2	CR[e]	330 (E)	> 99	
FBN	8	MB[d]	240 (M)	93	
FBN	2	ACF[c]	640 (M)	88.7	
FBN	2	CR[e]	600(M)	> 99	
FBN	2	EB[f]	560 (M)	> 99	

[a] Flux: the unit is L m^{-2} h^{-1}, [b] Rej: rejection, [c] ACF: Acid fuchsin, [d] MB: Methylene blue, [e] CR: Congo red, [f] EB: Evans blue. All flux data were normalized with 1 bar.

Supplementary Note 1. Concentration polarisation is described by the relationship between the solvent flux Jv, the bulk concentration C_B, the concentration at the wall of the membrane C_M, the mass transfer coefficient K, and the concentration in the permeate C_P as:

$$Jv = K \ln((C_M-C_P)/(C_B-C_P)) \qquad (1)$$

and the flux is described by:

$$Jv = B\,(AP-OP) \qquad (2)$$

where B = pure solvent permeance, AP = Applied Pressure and OP = Osmotic Pressure, and OP(from Van't Hoff equation) = c_M R T (3)

Therefore, there is an implicit equation in Jv governing flux when c_P is small relative to c_M and c_F

$$Jv-B(AP-C_M R\exp(Jv/K))=0 \qquad (4)$$

Supplementary References

1. Han, Y., Xu, Z. & Gao, C. Ultrathin graphene nanofiltration membrane for water purification. *Advanced Functional Materials* **23**, 3693-3700 (2013).

2. Huang, L. *et al.* Reduced graphene oxide membranes for ultrafast organic solvent nanofiltration. *Advanced Materials* **28**, 8669-8674 (2016).

3. Sun, L., Huang, H. & Peng, X. Laminar MoS_2 membranes for molecule separation. *Chemical Communications* **49**, 10718-10720 (2013).

4. Sun, L. *et al.* Ultrafast molecule separation through layered WS_2 nanosheet membranes. *ACS Nano* **8**, 6304-6311 (2014).

5. Zhang, R. *et al.* Coordination-Driven In Situ Self-Assembly Strategy for the Preparation of Metal–Organic Framework Hybrid Membranes. *Angewandte Chemie International Edition* **53**, 9775-9779 (2014).

6. Huang, H. *et al.* Ultrafast viscous water flow through nanostrand-channelled graphene oxide membranes. *Nature communications* **4**, 2979 (2013).

7. Ling, S., Qin, Z., Huang, W., Cao, S., Kaplan, D.L. and Buehler, M.J. Design and function of biomimetic multilayer water purification membranes. *Science Advances*, 3(4), p.e1601939 (2017).

8. Gao, S. J., Qin, H., Liu, P. & Jin, J. SWCNT-intercalated GO ultrathin films for ultrafast separation of molecules. *Journal of Materials Chemistry A* **3**, 6649-6654 (2015).

9. Ding, L. *et al.* A Two-Dimensional Lamellar Membrane: MXene Nanosheet Stacks. *Angewandte Chemie International Edition* (2017).

10. Karan, S., Jiang, Z. & Livingston, A. G. Sub–10 nm polyamide nanofilms with ultrafast solvent transport for molecular separation. *Science* **348**, 1347-1351 (2015).

11. Karan, S., Samitsu, S., Peng, X., Kurashima, K. & Ichinose, I. Ultrafast viscous permeation of organic solvents through diamond-like carbon nanosheets. *Science* **335**, 444-447 (2012).

12. Sun, S.-P., Chan, S.-Y., Xing, W., Wang, Y. & Chung, T.-S. Facile synthesis of dual-layer organic solvent nanofiltration (OSN) hollow fiber membranes. *ACS Sustainable Chemistry & Engineering* **3**, 3019-3023 (2015).
